# Risk Factors Associated With Human Papillomavirus Infection, Cervical Cancer, and Precancerous Lesions in Large-Scale Population Screening

**DOI:** 10.3389/fmicb.2022.914516

**Published:** 2022-06-30

**Authors:** Di Yang, Jing Zhang, Xiaoli Cui, Jian Ma, Chunyan Wang, Haozhe Piao

**Affiliations:** ^1^Department of Gynecology, Liaoning Cancer Hospital and Institute, Cancer Hospital of China Medical University, Shenyang, China; ^2^Department of Obstetrics and Gynecology, Shengjing Hospital of China Medical University, Shenyang, China; ^3^Department of Neurosurgery, Liaoning Cancer Hospital and Institute, Cancer Hospital of China Medical University, Shenyang, China

**Keywords:** cervical cancer, screening, high-risk factor, human papillomavirus infection, precancerous lesions

## Abstract

Cervical cancer is the most common gynecological malignancy and screening for risk factors with early detection has been shown to reduce the mortality. In this study, we aimed to analyze the characteristics and risk factors of human papillomavirus (HPV) infection and precancerous lesions in women and provide clinical evidence for developing strategies to prevent cervical precancerous lesions and cancer in women. Furthermore, we evaluated the influencing factors for high-risk HPV infection. From April 2018 to December 2021, 10,628 women were recruited for cervical cancer screening at Liaoning Cancer Hospital, Shenyang Sujiatun District Women’s and Infants Hospital, Benxi Manchu Autonomous County People’s Hospital, and Shandong Affiliated Hospital of Qingdao University. The study participants were tested to determine if they were HPV-positive (HPV +) or underwent thinprep cytology test (TCT) for atypical squamous cells of undetermined significance (ASCUS) and above. Furthermore, colposcopies and biopsies were performed for the histopathological examination. Finally, 9991 cases were included in the statistical analysis, and the factors influencing HPV infection and those related to cervical cancer and precancerous lesions were analyzed. HPV + infection, high-grade squamous intraepithelial lesion-positive (CINII +) in cervical high-grade intraepithelial neoplasia, and early cervical cancer diagnosis rates were 12.45, 1.09, and 95.41%, respectively. The potential risk factors for HPV were education ≤ high school [odds ratio (OR) = 1.279 (1.129–1.449), *P* < 0.001], age at initial sexual activity ≤ 19 years [OR = 1.517 (1.080–2.129), *P* = 0.016], sexual partners > 1 [OR = 1.310 (1.044–1.644), *P* = 0.020], ASCUS and above [OR = 11.891 (10.105–13.993), *P* < 0.001], non-condom contraception [OR = 1.255 (1.059–1.487), *P* = 0.009], and HSIL and above [OR = 1.541 (1.430–1.662), *P* < 0.001]. Compared with women aged 56–65 and 35–45 years [OR = 0.810 (0.690–0.950), *P* = 0.010] the HPV infection rate was significantly lower in those aged 46–55 years [OR = 0.79 (0.683–0.915), *P* = 0.002]. Furthermore, ≤ high school age [OR = 1.577 (1.042–2.387), *P* = 0.031], not breastfeeding [OR = 1.763 (1.109–2.804), *P* = 0.017], ASCUS and above [OR = 42.396 (28.042–64.098), *P* < 0.001] were potential risk factors for cervical cancer and precancerous lesions. In women with HPV infection, ≤ high school education level, initial sexual activity at ≤ 19 years of age, number of sexual partners > 1, ASCUS and above, non-condom contraception, HSIL and above were risk factors for HPV infection. Compared with women aged 56–65 years, those aged 35–45 and 46–55 years had significantly lower HPV infection rates, and high school age and below, non-breastfeeding, and ASCUS and above were all potential risk factors for cervical cancer and precancerous lesions.

## Introduction

Cervical cancer is the most common gynecological malignancy and in 2020, with 604,000 new cases worldwide, with approximately 342,000 related deaths (Sung et al., 2021). Cervical cancer has become the most common cancer in 23 countries and the leading cause of cancer-related deaths in 36 countries. The incidence rate of the disease in developing countries is significantly different from that in developed countries (Bray et al., 2018), with the highest occurring in sub Saharan Africa, South Polynesia, South America, and Southeast Asia (Sung et al., 2021). Inadequate living conditions and resources in developing regions, such as a lack of access to physical contraceptives and poor living and personal hygiene conditions, are considered factors that increase the burden of cervical cancer (Abulizi et al., 2017).

In contrast, developed countries have high coverage and availability of cancer screening and compliance by the target population (Thanapprapasr et al., 2012). The incidence of cervical cancer in China is not encouraging and the International Agency for Research on Cancer (IARC) reported 109,741 new cases in 2020. Furthermore, 59,060 patients died (Cao et al., 2021), accounting for 18.2 and 17.3% (Bray et al., 2018) of the cervical cancer incidence and death rate worldwide, respectively. According to the 2020 annual work report of the National Cancer Center, cervical cancer still has the sixth highest incidence of female cancers in China, and the mortality rate is still the eighth highest among female malignant tumors (Bray et al., 2018).

The incidence rate of cervical cancer at a younger age is showing an increasing trend (Zheng et al., 2019). Furthermore, cervical lesions are the most common diseases in women of childbearing age, and mainly include inflammation, injury, deformity, precancerous lesions, and tumors. Cervical cancer is usually caused by human papillomavirus (HPV) infection. HPV is a spherical DNA virus, which can cause proliferation of human skin mucosal squamous epithelium, and further reproduction may lead to various cervical diseases (McLaughlin-Drubin and Munger, 2008).

The German scholar Hausen first identified HPV infection as the main pathogenic factor of cervical cancer and precancerous lesions, which could be considered a milestone discovery in the prevention and treatment of this disease (Peter et al., 2010). A considerable amount of epidemiological and biological data have also proved that HPV infection is the main cause of cervical cancer and cervical intraepithelial neoplasia (Cordeiro et al., 2018). However, the HPV infection rate varies between regions because it is affected by numerous factors such as region, race, living habits, and HPV vaccination rate. Worldwide, economically developed regions such as South Korea, reported 18,170 women with HPV infection in 2014–2016, including 2,268 (12.5%) who were high-risk HPV positive (HPV +) (Ouh et al., 2018).

HPV E6/E7 mRNA detection is a new cervical cancer screening technology emerging in recent years. It is a screening method with E6/E7 as the detection target. Whether it has advantages in cervical cancer screening remains to be studied. The sensitivity of hpve6/E7 mRNA detection was lower than that of HR—HPV DNA detection, but the missed detection rate of hpve6/E7 mRNA detection was significantly lower than that of TCT and HR—HPV DNA detection (*P* < 0.05) (Giorgi Rossi et al., 2022).

The study of HPV prevalence and its subtype distribution may provide relevant information for routine vaccination and the types of HPV strains used in vaccination. HPV infection rates among women worldwide range from 11.70 to 7.20% (Lewandowska et al., 2021). The highest prevalence rates have been reported in Sub Saharan Africa (24.00%), Latin America and the Caribbean (16.10%), Eastern Europe (14.20%), and South East Asia (14.00%) (Bekmukhambetov et al., 2016). A study in the economically underdeveloped regions of West Africa showed that the high-risk HPV infection rate of 28.6% (Maria et al., 2018), whereas the overall HPV prevalence rate in Kazakhstan was reported to be as high as 43.8–55.8% (Aimagambetova and Azizan, 2018).

In China, significant differences were also reported in HPV infection rates in different regions, ranging from 13 to 31.9% (Zhong et al., 2017; Jiang et al., 2019). HPV also plays a vital role in the development of cervical lesions and cervical cancer, and the World Health Organization has confirmed that the mortality rate can effectively be reduced by screening. This study analyzed the characteristics and high-risk factors of HPV infection, cervical cancer, and high-grade precancerous lesions in a large-scale population screened for cervical cancer, to provide a reference for the prevention and treatment of these conditions.

## Patients and Methods

### Ethics Statement

This study was approved by the Ethics Committee of Liaoning Cancer Hospital and informed consent was obtain from all individual participants included in the study (No.: 20180106).

### Research Participants

From April 2018 to December 2021, we recruited 10,628 women for cervical cancer screening in Liaoning Province at Liaoning Cancer Hospital, Shenyang Sujiatun District Women’s and Infant Hospital, Benxi Manchu Autonomous County People’s Hospital, and Shandong Affiliated Hospital of Qingdao University. The average and median ages of the selected research participants were 49.61 ± 7.195 years, and 49 years, respectively.

### Methods

All women in the study underwent HPV testing and the thinprep cytology test (TCT). Community population and hospital outpatient opportunistic screenings were used to evaluate whether participants met the following inclusion criteria: (1) lived in Liaoning for > 3 years; (2) a history of sexual activity; (3) no sexual activity, vaginal medication, or drug flushing within 1 week before examination; (4) no serious organ dysfunction or mental illness; (5) voluntary participation and signed consent; and 5) willingness to complete the questionnaire survey. The exclusion criteria were: (1) women who were pregnant, lactating, or menstruating; (2) history of cervical surgery or hysterectomy; and (3) diagnosed with a tumor and being treated for other serious internal and external diseases. This study was approved by the ethics committee of Liaoning Cancer Hospital.

#### Sample Collection

##### Liquid-Based Cytology

TCT (Thinprep cytologic test purchased from Nanjing Xinbaishi Technology Co., Ltd.). The examination application forms of each patient was filled and their name and age and collection date was placed on the specimen bottle (liquid-based cytology preservation solution vial). The completely filled application form was checked to ensure the information was consistent with that on the preservation solution vial. The cervical brush was then placed in the preservation solution and rinsed by plunging it up and down 20 times, while spreading and rotating the bristles back and forth to make the cells fall into the solution.

Finally, the cervical brush was rapidly rotated to release the collected cells into the preservation solution, the sampler was discarded ensuring the brush head was not left in the bottle, and then it was sent to the pathology department for liquid-based analysis. The operating procedures of the system for the TCT, which is a liquid-based cytology production process, includes the following three steps, which were strictly adhered to: cell mixing, collection, and transfer. Briefly, the sample was placed into 95% alcohol for wet fixation, the production was completed, and then the next staining and diagnosis step was performed. The negative slides should be stored for 1 year, whereas the positive slides should be stored for a long time. Finally, the diagnosis was made by a full-time cytological diagnosis doctor in the pathology department.

##### Human Papillomavirus Detection Technology

The detection kit was purchased from Beijing Haolejie Healthcare Medical Equipment Company (Beijing). The E6/E7 mRNA detection kit was used to detect the following 14 high-risk HPV mRNAs known to cause cervical cancer (16, 18, 31, 33, 35, 39, 45, 51, 52, 56, 58, 59, 66, and 68), and displayed the detection results of 16 and 18 to provide more clinical guidance information. The kit detects E6 and E7 mRNA of high-risk HPV to avoid missing the detection of high-level lesions and cancer, which could result from only detecting the L1 region. Furthermore, no cross reaction occurred with low-risk HPV and there were fewer false positives, which reduced unnecessary colposcopies and over diagnosis.

#### Questionnaire and Survey

The questionnaire used in this study adopts the principle of voluntariness and was designed professionally by the members of our research group. The questionnaire includes questions on the participants’ personal information; relevant medical, reproductive, sexual activity, smoking, contraceptive, and sports history; educational level; and economic income.

#### Standard of Referral Colposcopy

Cervical exfoliative cells were examined using cervical liquid-based thinprep cytology test (TCT). The diagnostic criteria are based on the Bethesda system (TBS) classification: atypical squamous cells of undetermined significance (ASCUS) without clear diagnostic significance, excluding ACS-cannot exclude high-grade squamous intraepithelial lesion (ASC-H), low-grade squamous intraepithelial lesion (LSIL), high-grade squamous epithelial lesion (HSIL), squamous cell carcinoma (SCC), and atypical glandular epithelial cells (AGCs) (Nayar and Wilbur, 2015).

For HPV+, TBS classified ASCUS or above, or both, or clinically suspicious abnormalities, a colposcopy is further recommended. The examination results suggested the need for a multipoint tissue biopsy of cervical lesions and bite the tissue for pathological examination. The pathological results were the gold standard, and the diagnostic criteria included normal or inflammatory reaction and cervical intraepithelial neoplasia-intraepithelial neoplasia (CIN) and cervical cancer. CIN is divided into CIN I, CIN II, and CIN III (Kclurman et al., 2014) according to the following three levels: light, medium, and heavy, respectively.

#### Technical Quality Control

The quality control evaluation of the cervical exfoliative cell examination was conducted using 20 and 5–10% of the randomly selected positive and negative smears, respectively. All smears were reviewed by experts in the field and the acceptable quality rate of the smear results was 80%. The quality control of the colposcopy examination involved a spot check of 10 and 20% of the normal and abnormal reports, respectively. The results were rechecked by experts in the field and the standard quality rate of the reported results was expected to reach 90%. The quality control of the histopathological examination was performed by spot checking 10% of the pathological sections and the results were recheck by experts. The coincidence rate of diagnostic results was expected to reach 90%.

### Statistical Analysis

The statistical analysis of the relevant data was conducted using the statistical package for the social science (SPSS) version 19.0 software and the count data rate (%) was analyzed using the chi-square (χ^2^) test. The influencing factors were analyzed using univariate and multivariate logistic regression to evaluate the correlation between the relevant factors mentioned in the questionnaire and HPV infection, cervical cancer, and precancerous lesions.

## Results

### Characteristics of the Participants

#### Basic Information of Population

The patient age range was 35–65 years, and 1,271, 1,891, 2,788, 2,325, 1,536, and 807 were 35–40, 41–45, 46–50, 51–55, 56–60, and 61–65 year-old, respectively, corresponding to 11.95, 17.79, 21.88, 26.23, 21.88, 14.55, and 7.6% of the total population, respectively (Table 1).

**TABLE 1:** Basic information of cervical cancer screening population.

	Characteristics	N	*n* (%)
Age (years)	35–40	1,271	11.95
	41–45	1,891	17.79
	46–50	2,788	26.23
	51–55	2,325	21.88
	56–60	1,546	14.55
	61–65	807	7.60
Ethnicity	Han	8,932	84.04
	Others	1,696	15.9
Marital status	Unmarried	230	2.16
	Married	9,983	93.93
	Divorced	285	2.68
	Widowed	130	1.22
Profession	Head of party and enterprise unit	801	7.54
	Professional skilled worker	2,312	21.75
	Office and related personnel	1,137	10.70
	Social production and life service personnel	1,411	13.28
	Agriculture, forestry, animal husbandry, and fishery production and auxiliary personnel	368	3.4610
	Production and related personnel	1,107	10.42
	Soldier	8	0.08
	Others who are difficult to classify	2,647	24.91
	Others	837	7.88
Educational level	Junior high school and below	3,080	28.98
	Senior high school	3,341	31.44
	College degree or above	4,205	39.57
Total		10,628	100.0

#### Personal and Family History

The age of menarche and menopause for most participants was 12–18 years and > 50 years, respectively, accounting for 98.46 and 43.03%, respectively. Furthermore, 86.45% of participants had a history of breastfeeding and 89.42% had a cumulative breastfeeding time > 6 months. In addition, 5.92% of participant had multiple sexual partners and 2.27% had sex for the first time under the age of 19. The results also showed that 53.34% of participants had a history of miscarriage, whereas 3.67 and 4.10% reported prepuce and bleeding during intercourse, respectively, and a history of gynecological diseases and family history of tumor accounted for 12.82 and 10.11%, respectively (Table 2).

**TABLE 2:** Personal history and family history of cervical cancer screening population.

Characteristics		N	*n* (%)
Age at menarche (years)	<12	84	0.79
	12–18	10,464	98.46
	>18	80	0.75
Menopausal	Yes	4,573	43.03
	No	6,055	56.97
Age at menopause	<50	1,404	30.70
	≥50	3,169	69.30
Breastfeeding history	Yes	9,188	86.45
	No	1,440	13.55
Breastfeeding time	≤6 months	972	10.58
	>6 months	8,216	89.42
Sexual partners	0	15	0.14
	1	9,099	85.61
	≥2	629	5.92
Age at first sexual activity	Never	6	0.06
	≤19	242	2.27
	20–30	9,402	88.46
	≥31	91	0.86
Pregnancy history	Yes	9,981	98.61
	No	141	1.39
History of miscarriage	Yes	5,337	53.34
	No	4,668	46.66
Sexual partner’s foreskin is too long	Yes	372	3.67
	No	9,765	96.33
Bleeding during intercourse	Yes	412	4.1
	No	9,721	95.9
Cervical cancer vaccine	Yes	32	0.32
	No	10,099	99.68
Abnormal vaginal discharge	Yes	814	8.10
	No	9,237	91.90
Past history of gynecological disease	Yes	1,362	12.82
Family history of cancer	Yes	1,074	10.11

#### Participant Living Habits

Among the cervical cancer screening population, 56% were current smokers, 0.85% had quit smoking, 33.64% were passive smokers, and 80.46% were exposed to cooking fumes almost daily. In addition, 14.53 and 1.63% of the participants had a history of drinking and were still drinking, respectively, whereas 81.17% did not often participate in outdoor physical exercise and 91.49% did not drink tea. More than 60% of the participants had an insufficient intake of fresh vegetables and fruits, consuming > 5 catties/week and > 2.5 catties/week, respectively. Approximately 75% of human and animal meat intake does not meet the 50–100 g daily requirements of the Dietary Guidelines for Chinese Residents, as shown in Table 3.

**TABLE 3:** Living conditions of cervical cancer screening population.

Characteristics		N	*n* (%)
Smoking	No	9,935	93.49
	Currently smoking	602	5.66
	Smoking before	90	0.85
Secondhand smoke	Yes	3,575	33.64
	No	7,052	66.36
Cooking fumes	Almost everyday	8,550	80.46
	Sometimes	1,844	17.35
	Almost not	233	2.19
Alcohol consumption	No	8,910	83.84
	Currently drinking alcohol	173	1.63
	Previously drank alcohol	1,544	14.53
Exercise	Yes	2,107	19.83
	No	8,520	81.17
Tea drinking	Yes	904	8.51
	No	9,723	91.49
Vegetable consumption	Never eat	329	3.11
	<5 pounds/week	6,756	63.77
	≥5 pounds/week	3,509	33.12
Fruit consumption	Never eat	268	2.53
	<2.5 pounds/week	6,434	60.68
	≥2.5 pounds/week	3,902	36.78
Livestock meat consumption	Never eat	399	3.76
	≤350 g/week	7,841	73.95
	>350 g/week	2,363	22.29
Coarse grain consumption	Never eat	870	8.21
	<1 pounds/week	8,014	75.58
	≥1 pounds/week	1,719	16.21

#### Health and Emotional Status of Study Population

Among the cervical cancer screening population, 49.76, 8.64, 2.88, and 9.81% had very good or good health status, a history of hypertension, a history of diabetes, hyperlipidemia, mental illness, respectively. Furthermore, 22.75, 43.57, and 36.12% of the study population experienced recent negative life events, mental depression or anxiety symptoms, and poor sleep quality, respectively (Table 4).

**TABLE 4:** Health-related emotional factors of cervical cancer screening population.

Characteristics		N	*n* (%)
Self-assessed health status	Very good or good	5,288	49.76
	Generally	4,791	45.08
	Not good	548	5.16
Hypertension	Yes	918	8.64
	No	9,709	91.36
Diabetes	Yes	306	2.88
	No	10,321	97.12
Hyperlipidemia	Yes	1,042	9.81
	No	9,585	90.19
Diagnosed with a mental illness	Yes	27	0.25
	No	10,600	99.75
Experienced a negative life event	No	8,210	77.26
	1–2 piece	2,347	22.09
	3 pieces and above	70	0.66
Mental depression	No	5,997	56.43
	Occasionally	4,077	38.36
	>1 month	281	2.64
	>6 months	272	2.56
Anxiety	No	6,108	57.48
	Occasionally	4,066	38.26
	>1 month	286	2.69
	>6 months	167	1.57
Sleep quality	Good	6,788	63.88
	Hard to fall asleep	793	7.46
	Wake up early	1,266	11.91
	Sleep well	1,649	15.52
	Wake up at night	131	1.23
When you encounter difficulties, can get support from these people	Husband	9,294	87.46
	Parents	5,918	55.69
	Children	6,713	63.17
	Brothers and sisters	6,117	57.56
	Friends	5,302	49.89
	Colleagues	2,281	21.46
	No	80	0.75

#### Population Screening Willingness

Among the cervical cancer screening population, 16.54% thought they could easily develop cancer, whereas 27.15% had received cancer screening, and the cancer screening cost was completely covered by the government for up to 70.88% of the individual participants. In addition, > 78.28% of the study population expressed the willingness to fully accept cancer screening and the main reasons for not accepting cancer screening were time and no obvious symptoms.

Most participants were willing to undergo subsequent screening and could bear the expenses. The acceptable proportion of the out-of-pocket expenses was < 200 yuan in most instances. The data showed that 95.29% of the study population reported a willingness to return for a visit in case of abnormal results, and the main reason for those who were unwilling to was concerns that the examination might be painful. Furthermore, 87.1% of participants expressed a willingness to try a more effective new screening technology, with an acceptable out-of-pocket cost of < 200 yuan. The unwillingness to undergo new technology-based screening was mainly attributable to concerns that the interpretation and utilization of the screening results were unclear, as shown in Table 5.

**TABLE 5:** Screening willingness of cervical cancer screening population.

	Characteristics	N	*n* (%)
Do you think you are prone to cancer	Yes	1,738	16.54
	No	8,769	83.46
Have you ever been screened for cancer	Yes	2,885	27.15
	No	7,741	72.85
Who bears the cost of cancer screening	It is all borne by the government and not paid by individuals	1,979	70.88
	Some expenses shall be borne by individuals	492	17.62
	All expenses shall be borne by individuals	194	6.95
	No idea	127	4.55
To what extent do you accept cancer screening	Totally acceptable	2,166	78.28
	Acceptable	557	20.13
	Difficulty in accepting	31	1.12
	Unacceptable	13	0.47
Reasons for not participating in cancer screening	Economic reasons	2,362	22.23
	Time reason	4,425	41.64
	The procedure is cumbersome and laborious	3,244	37.4
	Examination can cause pain	3,409	30.53
	I do not think there are any symptoms in my body. It’s unnecessary	3,728	35.09
	Physical condition does not allow	81	0.76
	Unaccompanied	90	0.85
If the examination result is abnormal would you be willing to be checked again	Yes	9,298	88.06
	No	1,262	11.95
Would you like to be checked again	Yes	8,706	94.72
	No	485	5.28
What is the acceptable examination fee to you	<100 yuan	1,118	12.85
	100–199 yuan	4,037	46.39
	200–299 yuan	1,734	19.92
	≥300 yuan	1,814	20.84
Are you willing to make a return visit	Yes	9,333	95.29
	No	1,154	11.00
Reasons for not willing to make a return visit/recheck	Economic reasons	832	20.2
	Time reason	1,268	30.7
	The inspection is cumbersome and laborious	1,532	37.1
	Examination can cause pain	1,918	46.4
	I do not think there are any symptoms in my body. It is unnecessary	1,429	34.6
	Physical condition does not allow	58	1.4
	Unaccompanied	28	0.6
Are you willing to accept new technology	Yes	9,170	87.1
	No	1,360	12.9
How much are you willing to pay for the new technology at your own expense	<100 yuan	1,353	14.8
	100–199 yuan	4,410	48.3
	200,299 yuan	1,622	17.8
	≥300 yuan	1,736	19.0
Reasons for reluctance to accept new technology screening	Question the scientific validity and safety of the new method	1,748	38.9
	Unclear interpretation and utilization of screening results	2,548	56.7
	High cost	1,866	41.6
	The old method is reliable, there is no need to use the new method	731	16.3
	Concerned about the pain of new screening methods	432	8.8

### Human Papillomavirus and Thinprep Cytology Test Distribution

**FIGURE 1:**
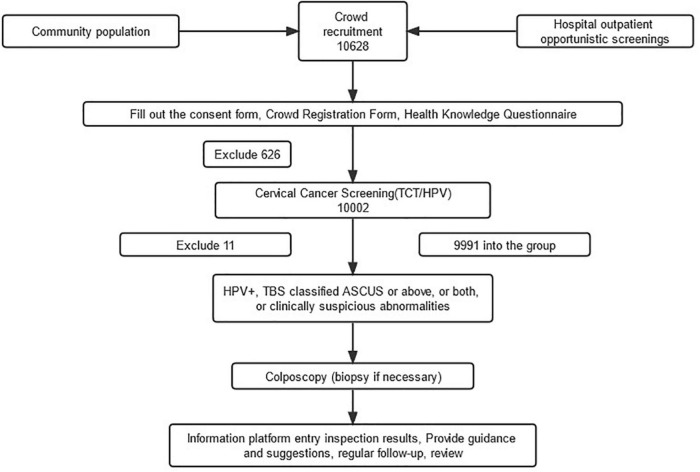
The CONSORT flow of study.

We recruited 10,628 women, aged 35–65 years, to participate in this study, including 626 who did not qualify and were subsequently excluded through the questionnaire survey results. Furthermore, 10,002 women were screened for cervical cancer, and 11 were subsequently excluded because they lacked a specimen and, thus, had no HPV examination results. Finally, 9991 participants were included in the statistical analysis, with an average and median age of 49.51 ± 7.188 and 49 years, respectively (Figure 1). The cytological examination result showed 721 TCT + cases, with 7.22% ASCUS + , which included 4.91% ASCUS, 0.49% ASC-H, 1.26% LSIL, 0.33% HSIL, 0.15% AGC-not otherwise specified (AGC-NOS), and 0.06% AGC-prone to cancer (AGC-N). In contrast, SCC, cervical carcinoma *in situ*, and adenocarcinoma were not detected (Table 6).

**TABLE 6:** Thinprep cytology test (TCT) cervical cancer screening results.

TBS classification diagnostic criteria	N	*n* (%)
NILM	9,271	9279
ASCUC	491	4.91
ASC-H	49	0.49
LSIL	126	1.26
HSIL	33	0.33
SCC	0	0.00
AGC-NOS	15	0.15
AGC-N	6	0.06
AIS	0	0.00
Adenocarcinoma	0	0.00
Total	9,991	100.00

*TBS, the Bethesda system; NILM, negative for intraepithelial lesion or malignancy; ASCUC, atypical squamous cells of undetermined significance; ASC-H, atypical squamous cells cannot exclude high-grade squamous intraepithelial lesion; LSIL, low-grade squamous intraepithelial lesion; HSIL, high-grade squamous intraepithelial lesion; SCC, squamous cell carcinoma; AGC-NOS, atypical glandular epithelial cells-not otherwise specified; AGC-N, atypical glandular epithelial cells-not prone to cancer; AIS, adenocarcinoma in situ.*

There were 1244 cases of HPV infection, and the positive detection rate was 12.45%. Among the HPV + women, 1213 were further tested for HPV typing, which showed that 184 (15.2%) and 65 (5.4%) were HPV 16 + and 18/45 + with detection rates of 1.84 and 0.65%, respectively. Furthermore, 980 (80.8%) patients were positive for other high-risk types of HPV, with a detection rate of 9.81%. Among the tested patients, 16 women had mixed HPV infections of the above three groups. The positive rate of HPV16 and HPV18/45 in the total screened population was 2.5% (247/9,991) (Table 7).

**TABLE 7:** Human papillomavirus (HPV) screening results in cervical cancer.

HPV		N	*n* (%)
Detection condition	(+)	1,244	12.45
	(−)	8,747	87.55
Typing	16 type	184	1.84
	18/45 type	65	0.65
	Other	980	9.81
Total		9,991	100.00

### Age Specificity of High-Risk Human Papillomavirus Infection

In this study, 9,991 participants were recruited for high-risk HPV screening in 2018–2021, which included 1,244 (12.45%) that were positive for high-risk HPV. The average age of the women was 49.99 (35–65) years and they were all divided into the following five age groups: 35–40, 41–45, 46–50, 51–55, and 56–65 years old. The prevalence of high-risk HPV infection had two peaks at 35–40, 55–60/61–65 years with infection rates of 13.09, 14.29, and 15.80%, respectively (Table 8).

**TABLE 8:** Age specificity of human papillomavirus (HPV) infection.

Age (years)	Total	HPV+	HPV–	Positive rate (%)	χ^2^	*P*
35–40	1,230	161	1,069	13.09	17.44	***0.0037***
41–45	1,792	201	1,591	11.22		
46–50	2,614	308	2,306	11.78		
51–55	2,192	254	1,938	11.59		
56–60	1,435	205	1,230	14.29		
61–65	728	115	613	15.80		

*Statistically significant (P < 0.05) values are indicated in bold.*

### Colposcopy

Further colposcopy was performed in 1,571 cases. There were 1,015 cases with complete colposcopy report, and the referral rate was 64.61%. There were 812 abnormal cases suspected to be diagnosed by colposcopy, 673 cases of low-grade lesions, and 66 cases of high-grade lesions, with a detection rate of 0.66% (Table 9).

**TABLE 9:** Colposcopy screening.

Initial diagnostic impression		N	*n* (%)	Detection rate (%)
Normal		203	20.00	
Abnormal	LSIL	673	66.31	6.74
	HSIL	61	6.01	0.61
	Suspected cancer	5	0.49	0.05
	Other	73	7.19	0.73
Total		1,015	−	−

### Single Factor Analysis of Risk Factors of Human Papillomavirus Infection

The results showed that 28 potential risk factors may be related to high-risk HPV infection. Univariate logistic analysis showed that HPV infection was correlated with different age groups, educational level, age of first sexual activity, number of sexual partners, contraceptive methods, and TCT positivity (*P* < 0.05) (Table 10).

**TABLE 10:** Comparison of different information and living habits with human papillomavirus (HPV) positive detection rate.

Characteristics		N	(+)	(−)	*n* (%)	χ^2^	*P*-value
Age (years)	35–45	3,022	362	2,660	11.98	15.04	***0.0009***
	46–55	4,806	562	4,244	11.69		
	56–65	2,163	320	1,843	14.79		
Family history of cancer	Yes	999	139	860	13.91	2.167	0.1410
	No	8,989	1,105	7,884	12.29		
Level of education	High school and below	6,040	815	5,225	13.49	15.08	***0.0001***
	College degree or above	3,947	429	3,518	10.87		
Age at initial sexual experience	≤19 year	236	42	194	17.8	5.817	***0.0154***
	>19 year	9,262	1,157	8,105	12.49		
Number of sexual partners	Multiple (≥ 2)	613	96	517	15.66	5.48	***0.0192***
	1	8,893	1,104	7,789	12.41		
Number of abortions	>1	2,209	279	1,930	12.63	1.119	0.2902
	1	2,970	405	2,565	13.64		
Number of deliveries	>1	1,043	138	905	13.23	0.6582	0.4172
	≤1	7,557	933	6,624	12.35		
Number of marriages	>1	406	55	351	13.55	0.3209	0.5710
	≤1	9,093	1,145	7,948	12.59		
Contraceptive methods used	Others	6,722	853	5,869	12.69	7.071	***0.0078***
	Condom	1,375	139	1,236	10.11		
Sexual partner has long foreskin	Yes	363	44	319	12.12	0.0456	0.8309
	No	9,537	1,192	8,345	12.50		
Bleeding during intercourse	Yes	405	56	349	13.83	0.7019	0.4022
	No	9,491	1,179	8,312	12.42		
Leucorrhea abnormality	Yes	795	88	707	11.07	1.627	0.2021
	No	9,018	1,139	7,879	12.63		
Vaccination	No	9,863	1,232	8,631	12.49	−	0.7909
	Yes	32	3	29	9.38		
Extramarital sex	Yes	66	11	55	16.67	1.055	0.3044
	No	9,830	1,226	8,604	12.47		
Ethnicity	Han	8,410	1,044	7,366	12.41	2.998	0.2234
	Man	1,431	175	1,256	12.23		
	Others	146	25	121	17.12		
Total household income	≤50,000 yuan	4,786	623	4,163	13.02	2.178	0.1400
	>50,000 yuan	4,181	501	3,680	11.98		
Menopausal	No	4,279	561	3,718	13.11	2.973	0.0846
	Yes	5,711	683	5,028	11.96		
Breastfed	No	1,312	163	1,149	12.42	0.001139	0.9731
	Yes	8,678	1,081	7,597	12.46		
Smoking history	Yes	619	68	551	10.99	1.303	0.2537
	No	9,371	1,176	8,195	12.55		
Alcohol consumption	Yes	1,599	198	1,401	12.38	0.008487	0.9266
	No	8,391	1,046	7,345	12.47		
Physical exercise	No	8,043	993	7,050	12.35	0.3278	0.5130
	Yes	1,947	251	1,696	12.89		
Tea drinking	No	9,145	1,144	8,001	12.51	0.3235	0.5695
	Yes	845	100	745	11.83		
Fresh vegetable consumption	No	6,594	811	5,783	12.30	0.4068	0.5236
	Yes	3,366	429	2,937	12.75		
Fresh fruit consumption	No	6,270	755	5,515	12.04	2.57	0.1089
	Yes	3,699	486	3,213	13.14		
Meat consumption	No	7,765	971	6,794	12.49	0.01798	0.8933
	Yes	2,202	273	1,929	12.40		
Coarse grain consumption	No	8,364	1,035	7,329	12.37	0.5294	0.4669
	Yes	1,604	209	1,395	13.03		
Are you in good health	No	504	71	433	14.09	1.301	0.2540
	Yes	9,486	1,173	8,313	12.36		
TCT	ASCUS and above	720	393	327	54.58	1,264	**<*0.0001***
	NILM	9,271	851	8,420	9.18		

*TCT, thinprep cytology test; ASCUC, atypical squamous cells of undetermined significance; NILM, negative for intraepithelial lesion or malignancy. Statistically significant (P < 0.05) values are indicated in bold.*

### Logistic Regression Analysis of Risk Factors of Human Papillomavirus Infection

The results of the multivariate unconditional logistic regression analysis showed that high school and below [odds ratio (OR) = 1.279 (1.129–1.449), *P* < 0.001], initial sexual activity age ≤ 19 years old [OR = 1.517 (1.080–2.129), *P* = 0.016], number of sexual partners > 1 [OR = 1.310 (1.044–1.644), *P* = 0.020], ASCUS and above [OR = 11.891 (10.105–13.993), *P* < 0.001], non-condom contraception [OR = 1.255 (1.059–1.487), *P* = 0.009], and HSIL and above [OR = 1.541 (1.430–1.662), *P* < 0.001] were risk factors for HPV infection. Compared with women aged 56–65 years, the HPV infection rate of those 35–45 [OR = 0.810 (0.690–0.950), *P* = 0.010] and 46–55 [OR = 0.79 (0.683–0.915)] years old (*P* = 0.002) decreased significantly (Table 11).

**TABLE 11:** Multivariate analysis of risk factors affecting human papillomavirus (HPV) infection.

Characteristics	SE	*P*-value	OR (95%)
35–45 year	0.082	0.010	0.810 (0.690∼0.950)
46–55 year	0.075	0.002	0.79 (0.683∼0.915)
High school and below	0.064	<0.001	1.279 (1.129∼1.449)
Initial age of sexual life ≤ 19 year	0.173	0.016	1.517 (1.080∼2.129)
Number of sexual partners > 1	0.116	0.020	1.310 (1.044∼1.644)
ASCUS and above	0.083	<0.001	11.891 (10.105∼13.993)
Contraceptive methods other than condoms	0.087	0.009	1.255 (1.059∼1.487)
HSIL and above	0.038	<0.001	1.541 (1.430∼1.662)

*OR, odds ratio; ASCUC, atypical squamous cells of undetermined significance; HSIL, high-grade squamous intraepithelial lesion.*

### Univariate Logistic Regression Analysis of Cervical Cancer and CIN II/III Univariate Analysis

Among the 9,991 patients included in the analysis, 1,004 had complete pathological results, including follow-up update, whereas 109 and 895 cases were CINII + and LSIL/inflammation, respectively. Furthermore, 136 and 1 patients were referred for a colposcopy because of TCT/HPV and clinically suspected abnormalities, respectively. The result of the colposcopy evaluation was negative and, therefore, no biopsy was performed. In addition, 137 patients were classified as LSIL and inflammation, and in the absence of pathological results, 8,210 women with double negative screening results (TCT-/HPV-) were regarded as having no cervical lesions. Therefore, 109 CINII + , 5 invasive carcinoma and 9, 242 LSIL/inflammation cases, respectively were included in the statistical analysis.

The detection rate of CINII + in cervical high-grade intraepithelial neoplasia was 1.09% (109/9,991), and the early diagnosis rate of cervical cancer was 95.41%(104/109). The results of the 28 logistic regression analyses using CINII + and diet as dependent variables showed a significant correlation between educational level, breastfeeding, and TCT positivity (*P* < 0.05). The detection rate of CINII + increased significantly with increasing age, early sexual activity, multiple sexual partners, total family income ≤ 50,000 yuan, bleeding during sexual intercourse and extramarital sex, but there was no significant difference (Table 12).

**TABLE 12:** Comparison of detection rates of high-grade squamous intraepithelial lesion positive (CINII +) in populations with different basic characteristics.

Characteristics		N	CINII +	LSIL/inflammation	*n* (%)	χ^2^	*P*-value
Age (years)	35–45	2,804	30	2,774	1.07	1.76	0.4147
	46–55	4,542	50	4,492	1.10		
	56–65	2,005	29	1,976	1.45		
Family history of cancer	Yes	952	10	942	1.05	0.06594	0.7973
	No	8,396	96	8,300	1.14		
Level of education	High school and below	5,657	77	5,580	1.36	4.727	***0.0297***
	College degree or above	3,690	32	3,658	0.87		
Age at initial sexual activity	≤19 year	211	3	208	1.42	−	0.7410
	>19 year	8,688	103	8,585	1.19		
Number of sexual partners	>1	567	9	558	1.59	1.177	0.2780
	≤1	8,339	91	8,248	1.09		
Number of abortions	>1	2,088	32	2,056	1.53	0.1755	0.6753
	≤1	2,810	39	2,771	1.39		
Number of deliveries	>1	966	13	953	1.35	0.1977	0.6566
	≤1	7,120	84	7,036	1.18		
Number of marriages	>1	383	7	376	1.83	1.378	0.2405
	≤1	8,516	99	8,417	1.16		
Contraceptive methods	Others	6,332	75	6,257	1.18	0.08034	0.7768
	Condom	1,283	14	1,269	1.09		
Sexual partner has long foreskin	Yes	341	3	338	0.88	−	>0.9999
	No	8,926	104	8,822	1.17		
Bleeding during sexual intercourse	Yes	373	7	366	1.88	1.843	0.1746
	No	8,891	99	8,792	1.11		
Leucorrhea abnormality	Yes	738	10	728	1.36	0.2504	0.6168
	No	8,443	97	8,346	1.15		
Vaccination	No	1,125	106	1,019	9.42	−	>0.9999
	Yes	3	0	3			
Extramarital sex	Yes	63	2	61	3.17	−	0.1646
	No	9,198	105	9,093	1.14		
Ethnicity	Han	7,860	93	7,767	1.18	0.3176	0.8532
	Man	1,350	14	1,336	1.04		
	Others	137	2	135	1.46		
Total household income	≤50,000 yuan	5,247	65	5,182	1.24	3.526	0.0604
	>50,000 yuan	3,856	32	3,824	0.83		
Menopausal	No	5,334	62	5,272	1.16	0.001261	0.9717
	Yes	4,016	47	3,969	1.17		
Breastfed	No	1,240	23	1,217	1.85	5.891	***0.0152***
	Yes	8,110	86	8,024	1.06		
Smoking history	Yes	583	9	574	1.54	0.7709	0.3799
	No	8,767	100	8,667	1.14		
Alcohol consumption	Yes	1,476	18	1,458	1.22	0.04393	0.8340
	No	7,874	91	7,783	1.16		
Physical exercise	Yes	1,836	18	1,818	0.98	0.6814	0.4091
	No	7,514	91	7,423	1.21		
Tea consumption	No	8,567	101	8,466	1.18	0.1539	0.6948
	Yes	783	8	775	1.02		
Fresh vegetable consumption	No	6,124	66	6,058	1.08	1.302	0.2538
	Yes	3,196	43	3,153	1.35		
Fresh fruit consumption	No	5,851	67	5,784	1.15	0.07296	0.7871
	Yes	3,479	42	3,437	1.21		
Meat consumption	No	7,265	85	7,180	1.17	0.0007221	0.9786
	Yes	2,064	24	2,040	1.16		
Coarse grain consumption	No	7,827	91	7,736	1.16	0.01458	0.9039
	Yes	1,501	18	1,483	1.20		
Are you in good health	No	464	4	460	0.86	0.3908	0.5319
	Yes	8,886	105	8,781	1.18		
TCT	ASCUS and above	513	74	439	14.42	828.3	**<*0.0001***
	NILM	8,838	35	8,803	0.40		

*TCT, thinprep cytology test; ASCUC, atypical squamous cells of undetermined significance; NILM, negative for intraepithelial lesion or malignancy. Statistically significant (P < 0.05) values are indicated in bold.*

### Multivariate Logistic Regression Analysis of Cervical Cancer and CIN2/3 Risk Factors

The factors that were statistically different in the univariate unconditional logistic regression analysis were further analyzed using multivariate unconditional logistic regression. The results showed that high school and below [OR = 1.577 (1.042–2.387), *P* = 0.031], not breastfeeding [OR = 1.763 (1.109–2.804), *P* = 0.017], ASCUS and above [OR = 42.396 (28.042–64.098), *P* < 0.001] were potential risk factors for cervical cancer and precancerous lesions (Table 13).

**TABLE 13:** Multivariate analysis of risk factors for cervical cancer and precancerous lesions.

Characteristics	SE	*P*-value	OR (95%)
High school education and below	0.211	0.031	1.577 (1.042∼2.387)
Not breastfeeding	0.237	0.017	1.763 (1.109∼2.804)
ASCUS and above	0.211	<0.001	42.396 (28.042∼64.098)

*SE, standard error; OR, odds ratio; ASCUC, atypical squamous cells of undetermined significance.*

## Discussion

In this multicenter, cross-sectional population study in women, we found that the overall prevalence of HR-HPV was 12.45%, which is lower than the national average. HPV infection can occur at any age and is related to age. In this study, the age-specific distribution showed a bimodal curve, and the first peak appeared in the 35–40-year-old age group, with the infection rate of middle-aged women showing a low trend. The second peak of high-risk HPV infection was observed in women aged 55–60 and 61–65 years, who were born in the 1960s–1970s and the economy of China has improved considerably since then. In addition, the education level of these age groups is significantly lower than that of the younger women, which contributes to their lack of knowledge about HPV infection. In addition, the low level of autoimmunity and hormones further impairs the resistance of the cervix to HPV infection, which may explain the significant increase in their HPV + rate.

Presently, there is no specific or effective drug treatment for HPV infection, and although a vaccine has been developed and listed in China, it is expensive and does not prevent all subtype infections (You et al., 2020). Therefore, the prevention of HPV infection is very important. Number studies have reported the following as some factors to be related to the incidence of cervical cancer: sexual activity, early first sexual activity, premature delivery, prolificacy, high-risk HPV infection, and smoking. The following are some of the factors related to cervical intraepithelial neoplasia: sexual activity, HPV infection, smoking, premature sexual activity, sexually transmitted diseases, low economic status, use of oral contraceptives, and immunosuppression (Shannon et al., 2017).

In this study, multicenter cervical screening was used to analyze the incidence of HPV infection, cervical cancer, and high-grade precancerous lesions in women. The results led us to conclude that high school and below, initial sexual life age ≤ 19 years old, number of sexual partners > 1, ASCUS and above, non-condom contraception, HSIL and above were all risk factors of HPV infection. The reason may be that women with a low educational level lack the awareness of cervical screening and prevention strategies (Williams et al., 2019). Women ≤ 19 years old who are sexually active are prone to HPV infection because of their immature cervical development and incompletely developed autoimmune function (Morris et al., 2019). Furthermore, a higher number of sexual partners increases the potential exposure to HPV infection, rendering an individual prone to HPV infection.

Numerous studies have shown that an active sex life is closely related to the occurrence and development of cervical cancer. Literature reports from countries other than China state that factors such as sexual partners and frequency are closely related to cervical cancer (Torres-Poveda et al., 2019). Condoms block pathogens from damaging the cervical mucosa and inhibiting the immune function, whereas cleaning the vulva reduces the probability of infection. Both processes reduce the stimulatory effects of semen on the cervical mucosa, which is consistent with the research results of Hariri and Warner (2013) on male condoms that indicates that they contribute to reducing the transmission of HPV. The incidence of CINII and above was positively correlated with HPV infection.

Cervical lesions mostly occur in married women and it is the most serious cervical disease, and shows higher occurrence in younger women (Arbyn et al., 2020). Cervical cancer, which is mainly caused by long-term cervical lesions, has a high mortality, which is gradually increasing (Arbyn et al., 2020). Therefore, preventing cervical lesions is extremely significant for women (Cree et al., 2021). HPV infection has always been considered an important factor in the development of cervical lesions, but most women can be protected by the autoimmune system. In addition, a few women will continue to be infected and develop cervical cancer over time. Therefore, the need to actively understand cervical lesions, HPV infection, and the influencing factors has been increasingly attracting clinical attention. Studies suggest that high-risk HPV infection was detected in 99.7% of cervical cancer patients (Crosbie et al., 2013).

HPV has been reported to be transmitted through direct or indirect skin or sexual contact (Wierzbicka et al., 2022). The infection occurs worldwide with approximately 4–20% of healthy individuals harboring the infection, and the cumulative lifetime infection rate is 60–70%. Sustained expression of the E6 and E7 HPV virus subtypes has been found to be closely related to the growth of HPV-infected cancer cells (Pinatti et al., 2017). HPV infection is the most important and definite cause of cervical cancer (Araldi et al., 2018). In this study, we found that an increasing histopathological grade of HR-HPV infection was associated with the incidence of cervical cancer. The results showed that a high school education and below, non-lactation, and TCT positivity were statistically significant (*P* < 0.05) risk factors of cervical cancer and CIN II/III.

The CINII + detection rate increased with increasing age, early sexual activity, multiple sexual partners, total family income ≤ 50,000 yuan, bleeding during sexual intercourse, and extramarital sex, but not significantly. This study has been compared with other similar related research data in the near future (see Supplementary Table 1 for details) (Kitamura et al., 2021; Tagne Simo et al., 2021; Niu et al., 2022). As a preventable malignant tumor, the occurrence and development of cervical cancer is a gradual process that takes several years to progress from intraepithelial lesions to invasive disease. Effective screening methods for cervical cancer could ensure early detection, diagnosis, and treatment of cervical precancerous lesions and, to a certain extent, reduce its incidence rate and mortality.

## Conclusion

Cervical cancer is a prominent public health problem and proms focusing on its prevention and control should be strengthened. In this study, multicenter cervical screening of women to determine the HPV infection rate and incidence of cervical high-grade precancerous lesions, demonstrated a high school education and below, initial age of sexual activity ≤ 19 years old, more than one sexual partner, ASCUS and above, non-condom contraception, and HSIL and above as risk factors of HPV infection. Furthermore, a high school education and below, non-lactation, and a positive TCT result were identified as risk factors of cervical cancer and high-grade precancerous lesion infection. In conclusion, the investigation of risk factors for HPV infection and cervical high-grade precancerous lesions are of great significance for reducing the incidence rate of cervical cancer. Consequently, we recommend the establishment of strategies to enhance the awareness of the risk factors of high-risk HPV infection; to promote healthy sexual behavior, life, and health habits; and to improve immunity and strengthen physical exercise, which could effectively reduce the incidence rate of cervical cancer.

## Data Availability Statement

The raw data supporting the conclusions of this article will be made available by the authors, without undue reservation.

## Ethics Statement

The studies involving human participants were reviewed and approved by the Ethics Committee of Liaoning Cancer Hospital (No. 20180106). The patients/participants provided their written informed consent to participate in this study. Written informed consent was obtained from the individual(s) for the publication of any potentially identifiable images or data included in this article.

## Author Contributions

HP and CW designed and supervised the project. DY, JZ, and XC collected clinical data samples. DY collected and processed data and data analysis, and drafted the manuscript. JM translated and polished the manuscript. All authors reviewed, discussed, and edited versions of the final report manuscript.

## Conflict of Interest

The authors declare that the research was conducted in the absence of any commercial or financial relationships that could be construed as a potential conflict of interest.

## Publisher’s Note

All claims expressed in this article are solely those of the authors and do not necessarily represent those of their affiliated organizations, or those of the publisher, the editors and the reviewers. Any product that may be evaluated in this article, or claim that may be made by its manufacturer, is not guaranteed or endorsed by the publisher.
