# Supplementary material for: Risk Factors Associated With Human Papillomavirus Infection, Cervical Cancer, and Precancerous Lesions in Large-Scale Population Screening
Source: Front Microbiol. 2022 Jun 30;13:914516. doi: 10.3389/fmicb.2022.914516 (PMC9282163; doi:10.3389/fmicb.2022.914516)
Supplement: Supplementary file 1 [file Table_1.docx]

Supplementary Table 1. Comparison of Similar Data

| Recent similar data | HR-HPV positive rate(%) | Risk factors for HPV (+) | Risk factors for CINII(+) |
| --- | --- | --- | --- |
| This research | 12.45 | education ≤high school | ≤high school |
|  |  | age at initial sexual activity ≤19 years | not breastfeeding |
|  |  | sexual partners >1 | ASCUS and above |
|  |  | ASCUS and above | - |
|  |  | non-condom contraception | - |
|  |  | HSIL and above | - |
| Literature 1^a^ (Shanghai China) | 9.5 | education level | - |
|  |  | type of vaginitis | - |
|  |  | history of hyperlipidemias | - |
|  |  | family history of cancer | - |
|  |  | number of pregnancies | - |
|  |  | number of sex partners | - |
| literature 2^b^ (Japan) | 28.1 | The number of lifetime sex partners (≥6) | - |
|  |  | present history of sexually transmitted infection | - |
| literature 3^c^ (Cameroon) | 38.7 | - | the number of sexual partners |
|  |  | - | history of oral contraceptive pill use |

1. [Niu J, Pan S, Wei Y, Hong Z, Gu L, Di W, et al. Epidemiology and analysis of potential risk factors of high-risk human papillomavirus (HPV) in Shanghai China: A cross-sectional one-year study in non-vaccinated women. J Med Virol. 2022 Feb;94(2):761-770.PMID: 34766625].
2. [Kitamura T, Suzuki M, Shigehara K, Fukuda K. Prevalence and Risk Factors of Human Papillomavirus Infection among Japanese Female People: A Nationwide Epidemiological Survey by Self-Sampling. Asian Pac J Cancer Prev. 2021 Jun 1;22(6):1843-1849.PMID: 34181341].
3. [Tagne Simo R, Djoko Nono AG, Fogang Dongmo HP, Seke Etet PF, Fonyuy BK, Kamdje AHN, et al. Prevalence of precancerous cervical lesions and high-risk human papillomavirus types in Yaounde, Cameroon. J Infect Dev Ctries. 2021 Sep 30;15(9):1339-1345.PMID: 34669605].
